# COVID-19: where is the national ethical guidance?

**DOI:** 10.1186/s12910-020-00478-2

**Published:** 2020-05-01

**Authors:** Richard Huxtable

**Affiliations:** Centre for Ethics in Medicine, Population Health Sciences, Bristol Medical School, Canynge Hall, 39 Whatley Road, Bristol, BS8 2PS UK

**Keywords:** COVID-19, Coronavirus, Ethical guidance, Professional guidance

## Introduction

The COVID-19 pandemic poses numerous – and substantial – ethical challenges to health and healthcare. Debate continues about whether there is adequate protective equipment, testing and monitoring, and about when a vaccine might become available and social restrictions might be lifted. The thorny dilemmas posed by triage and resource allocation also attract considerable attention, particularly access to intensive care resources, should demand outstrip supply.

But the “COVID fog” clouds more than the intensive care unit [1]. The provision and uptake of non-COVID related treatment is declining, due to the de-prioritisation of some services and interventions, alongside non-COVID patients’ fears of contracting the virus; difficult conversations are being held in suboptimal circumstances; and final farewells and death rituals have been disrupted. Healthcare personnel, meanwhile, are facing moral distress and, for some, difficulties arising from undertaking new roles in unfamiliar settings.

Whilst patients and the public require support, health and social care professionals also need guidance to help navigate the ethical challenges. A pandemic (by definition) respects no geographical boundaries, so co-ordinated international efforts will be important [2]. But guidance will also be needed to inform decision-making in health and social care within and throughout countries.

Focusing on the UK, the Scottish Government has commendably issued a national ethics framework [3]. For its part, the government in England has expanded intensive care capacity, via new Nightingale hospitals, which might reduce or remove the need to engage with the more contested questions of resource allocation [4]. However, to date, there is no authoritative ethical guidance in England that can help professionals to answer those questions, or indeed the many others arising. That vacuum now needs to be filled.

## The need for clarity, consistency and fairness

Authoritative national ethical guidance should help to bring clarity, consistency and fairness to decision-making.

Although authoritative guidance is lacking in England, influential and respected professional organisations, like the Royal College of Physicians [5], the British Medical Association (BMA) [6], and other Royal Colleges, are issuing (explicitly) “ethical” guidance [7, 8]. Ethical guidance is also being prepared at the regional and local levels. Other guidance is also emerging, which does not explicitly talk of ethics, but which nevertheless engages with inherently ethical questions, such as from NICE, on critical care during COVID-19 [9], and from NHS England, on hospital admissions for older patients [10].

The latter are authoritative bodies, to which health and social care professionals must listen. Unfortunately, as we will see below, their guidance has not been uncontroversial. Also troubling is the fact that the guidance landscape in England is fast becoming difficult to navigate. Although, within the bounds of confidentiality, efforts are being made to share approaches, the proliferation of guidance from various sources and its frequent updating risks either contradiction or duplication of effort. Rather than being guided, professionals confront information-overload, which might leave them distrustful of issuing agencies and, fundamentally, unsure of which guidance to follow and, correspondingly, of their obligations.

Such confusion increases the risk of inconsistency. Formal justice requires that like cases be treated alike; as Aristotle instructed, equals should be treated equally, and unequals unequally [11]. The Scottish guidance rightly seeks to strike a balance, recognising the need for a uniform framework, whilst also implying that there might be valid distinctions to be drawn between the needs and interests of different groups, individuals, settings and regions. In addition to offering (high-level) principles, the document accordingly points to local clinical ethics support services, which can presumably offer ethical support that accounts for local needs [3].

Of course, whilst some local variation might be legitimate, “postcode lotteries” appear unjust. Unfortunately, neither justice nor related terms (like fairness) are easy to pin down. When is discrimination between people just and when is it unjust? And which processes are required to ensure that justice is done – and seen to be done?

Emerging guidance in England has been charged with failing adequately to answer such questions. NICE and NHS England guidance has been criticised – and consequently re-drafted – for making unjustly discriminatory judgments about older individuals or those with disabilities and co-morbidities [12, 13]. No doubt mindful of such challenges, the BMA has sought to supplement its ethical guidance, acknowledging that the age and pre-existing health condition of a patient with COVID-19 may be pertinent to their “ability to benefit” from (for example) intensive care, but that blanket exclusions based on age or disability “would be both unacceptable and illegal” [14].

This, of course, raises the question of what a just or fair allocation of scarce resources would look like. Difficult substantive questions obviously arise: which account of distributive justice should inform decisions, and thus determine which patients should receive – or not receive – (even life-saving) treatment? There are also significant procedural questions: who should be involved in making or contributing to such decisions, especially during a fast-moving pandemic?

## The need for guidance

As difficult as these questions are, professionals – and patients – are understandably looking for information and support and, fundamentally, for answers. Current efforts to provide these are commendable. But clarity, consistency and fairness may best be served by authoritative national ethical guidance.

The task will not be easy, and guidance will need to ensure that it hears and heeds the voices of stakeholders, including health and social care professionals, as well as patients, all of whom might otherwise resent the imposition. Consultation may be challenging during a pandemic, but it should not be impossible.

Without wishing to presume what such guidance might ultimately say, three observations may be made. First, following the Scottish example, authoritative guidance could usefully outline the “headline” principles, which should guide decision-making. England already has some such principles, first developed in relation to pandemic flu in 2009, which should offer a useful starting point [15].

Of course, as the Scottish guidance also illustrates, high-level principles might not provide the requisite steer: for example, that document notes the need to “consider fairness of healthcare distribution” ([3], p. 4), but this obviously requires specification. Second, then, more detailed guidance will be needed to “cash out” the principles in relation to the decisions that will, or are likely to, lie ahead. Third, provision should also be made for more localised decision-making, at least where there may be a legitimate need for variation or context-sensitivity; clinical ethics committees may be helpful here.

In sum, a hub-and-spokes model is conceivable, which aims to offer clarity, consistency and fairness, by advancing guiding principles and explaining how these might apply to different decisions, locales, professionals and patients. As the BMA notes, healthcare professionals deserve and “need support and clear guidance in advance to make sure … decisions are being made in a fair and consistent way” [[6], p. 1]. Patients, too, want clarity: a legal case has begun in England, challenging “the lack of a national framework for treatment prioritisation, if demand for life-sustaining treatment outstrips supply during the COVID-19 pandemic” [16]. The outcome may be salutary, not only in England, but also in other countries that lack an authoritative national lead. Of course, triage and allocation are not the only ethical questions at this very difficult time. On these – and all the other questions posed by and within the “COVID fog” – clear, consistent and fair guidance is needed.

## Data Availability

Not applicable.
